# A Lanthanide-Based Chemosensor for Bioavailable Fe^3+^ Using a Fluorescent Siderophore: An Assay Displacement Approach

**DOI:** 10.3390/s100201326

**Published:** 2010-02-11

**Authors:** Karen M. Orcutt, W. Scott Jones, Andrea McDonald, David Schrock, Karl J. Wallace

**Affiliations:** 1 Department of Marine Science, University of Southern Mississippi, 1020 Balch Blvd. Stennis Space Center, MS 39529, USA; 2 Department of Chemistry and Biochemistry, University of Southern Mississippi, 118 College Drive, Hattiesburg, MS 39406, USA; E-Mail: Karl.Wallace@usm.edu (K.J.W.)

**Keywords:** lanthanide, desferrioxamine, siderophore, iron, chemosensor, indicator displacement assay

## Abstract

The measurement of trace analytes in aqueous systems has become increasingly important for understanding ocean primary productivity. In oceanography, iron (Fe) is a key element in regulating ocean productivity, microplankton assemblages and has been identified as a causative element in the development of some harmful algal blooms. The chemosenor developed in this study is based on an indicator displacement approach that utilizes time-resolved fluorescence and fluorescence resonance energy transfer as the sensing mechanism to achieve detection of Fe^3+^ ions as low as 5 nM. This novel approach holds promise for the development of photoactive chemosensors for ocean deployment.

## 1. Introduction

Currently, there is a need for ultra-sensitive, real-time monitoring and detection technologies in marine science. The measurement of trace analytes in aqueous systems has become increasingly important for understanding the controls of ocean primary productivity. In oceanography, iron (Fe) (total iron *i.e.*, Fe^2+^ and Fe^3+^) is a key element in regulating the efficiency of the biological pump, the CO_2_ absorbing mechanism of the ocean [1]. The development of a sensitive photoactive chemosensor for biologically available Fe provides a model to build sensors for other biologically relevant analytes, such as other trace metals.

Biologically available Fe is the form that is nutritionally available to microorganisms. The majority of this fraction of Fe in seawater is coordinated to organic ligands. The fungal siderophore Desferrioxamine B (DFB) specifically complexes Fe over all other bioactive metals and can extract Fe from seawater [2–4]. DFB has a high binding affinity (log K ∼30.6) for Fe^3+^ and when added to natural seawater, it dramatically decreases the ability of microplankton to take up Fe [2–4]. Thus, DFB binds Fe that was previously nutritionally available to microplankton. This is the fraction of Fe that will be the target analyte in this chemosensor application and the DFB molecule will serve as the reactive interface.

Elemental Fe is a key regulator in oceanic primary productivity, microbial community assemblages and has been identified as an agent in the development of some harmful algal blooms. Although there are several methodologies to measure total Fe in seawater, there are few analytical methods for measuring ultra-trace biologically available Fe fraction in seawater [5]. Recently, novel approaches have been developed for assessing biologically available Fe in aqueous systems. For example, bioreporters, engineered cells that emit light under iron deficiency, are a direct approach to assessing the bioavailability of iron to heterotrophic bacteria and cyanobacteria [6–14]. Bioreporters were first demonstrated in fresh water systems using engineered bacterial and cyanobacterial cells [7,12] but they also have been used in marine systems to measure biologically available iron [6,11,15]. While these systems provide a measure of biologically available iron to prokaryotes, they cannot provide a measure of biologically available iron to eukaryotic phytoplankton. Another approach to determine the bioavailability of iron is to monitor the fluorescent signal using encapsulated bacterial parabactin sensor. This approach uses flow cell technology equipped with a sol-gel film with detection limits as low as 40 pM [16]. It takes approximately 20 minutes for a measurement-regeneration cycle. Another approach miniaturized a bulk liquid membrane system to a liposome-based nanodevice to sequester siderophore bound iron, demonstrating the feasibility of a nanostructured approach [17].

Fluorescence detection of Fe in cells and biological fluids use fluorescent siderophores [18,19]. These molecules can be fluorescent bacterial siderophores such as parabactin or azotobactin, DFB derivatives and reversed siderophores [18]. Fluorescent DFB siderophores are produced by a bioconjugate technique that links a fluorophore group to the amine group on the pendant arm of DFB. A chemically derived DFB siderophore called *N*-Methyanthranyl desferrioxamine (MA-DFB) has been used by Palanche *et al.* and has shown promise as a possible environmental chemosensor in natural waters with a reported detection limit of 1.1 ng/mL [19].

This paper presents preliminary evidence that a fluorescent siderophore can be used to sensitize a lanthanide ion, that is displaced upon the addition of Fe(III). The initial studies show that the present study has lowered the detection limit to 0.28 ng/mL in relation to previously reported systems, thus increasing the sensitivity at least four fold.

## 2. Results and Discussion

### 2.1. Design Criteria

**Figure 1.**
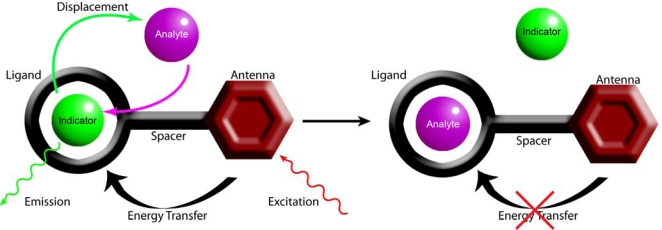
Cartoon representation of the Indicator Displace Assay (indicator = lanthanide, analyte = Fe^3+^ antenna = *N*-Methyanthranyl moiety).

Indicator displacement sensing, also referred to as an indicator displacement assay (IDA), has become an attractive method to detect analytes [20–23]. In a typical IDA approach, the indicator competes with the substrate for the same binding site via rapid reversible interactions. The indicator sits within the cavity of the receptor, with a particular λ_max_, indicative of the microenvironment of the receptor. Upon addition of a guest, the indicator is displaced into free solution and an alternative wavelength is observed (Figure 1). As with any host–guest system design, there needs to be careful consideration of the target guest. Factors such as shape, size, charge, and hydrogen bonding donor/acceptor characteristics need to be taken into account. The binding sites for the particular guest need to be complimentary to the binding sites of the host. The more favorable the interactions, the more stable the host–guest complex.

**Scheme 1.**
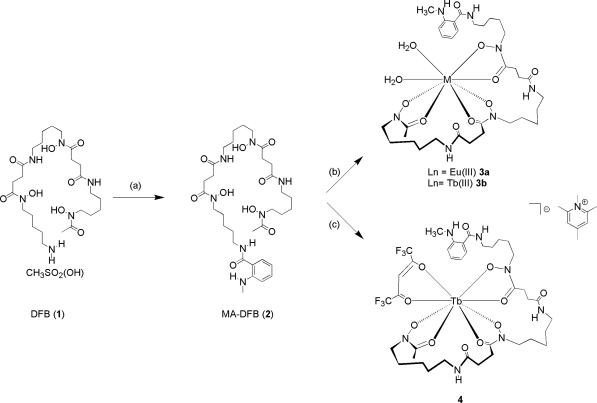
Synthesis of MA-DFB-Ln(III) (a) N-methylisatoic anhydride and Et_3_N (b) LnCl_3_•6H_2_O (Ln = Eu(III) or Tb(III), 2,4,6 trimethylpyridine (c) TbCl_3_•6H_2_O, 1,1,1,5,5,5-hexafluoro-2, 4-pentanediione (Hhfac) and 2,4,6 trimethylpyridine.

The host-guest system described in this paper is such that the indicator of choice is a lanthanide ion (Eu^3+^ or Tb^3+^) coordinated to MA-DFB (compound **2**- the host). It is well known that lanthanide complexes undergo rapid ligand exchange [24]. Lanthanide^3+^ ions are hard Lewis acids that have a tendency to form stable complexes with hard Lewis bases, those that contain oxygen atoms. Therefore the nature of the bonding in these complexes is predominately ionic and highly stable. However, lanthanide complexes in general, are also kinetically labile [25]. This is in contrast to the ferric ion siderophore coordination species whereby the complex is thermodynamically stable and kinetically inert. Taking this into consideration, the rationale behind the sensor design described in this paper was to utilize and synthesize an ID sensor, whereby the host-indicator (Ln-MA-DFB) was prepared (scheme 1) and Fe^3+^ acting as the guest.

It is well known that lanthanide ions, in particular Eu(III) and Tb(III) salts, have poor luminescence properties, due to *f-f* forbidden transitions, and as a consequence, lanthanide ions are not usually excited directly. Instead, they require a fluorophore (antenna) attached to a chelating functionality that incorporates the lanthanide metal [26,27]. The fluorophore (known as a sensitizer molecule) is typically an organic functional group. The organic fluorophore acts as an antenna absorbing light and transferring energy (*via* fluorescent transfer) to the lanthanide ion. An advantage of using lanthanide ions as part of a sensing application is their well-defined narrow excitation and emission bands and their long (micro- to millisecond) fluorescence, making them excellent candidates for molecular sensors. The long lifetimes (>200 μs) of Ln luminescence allow for discrete signal detection without background fluorescence, providing temporal selectivity for the lanthanide ion.

The advantage of this type of host-guest receptor is the sensing mechanism employed in the system, being a combination of Time-Resolved Fluorescence (TRF) and Fluorescent Resonance Energy Transfer (FRET). This union allows for greater analytical sensitivity because of the use of rare earth ions (lanthanides). A more correct description of energy transfer using lanthanides is Lanthanide Resonance Energy Transfer (LRET). LRET has a number of technical advantages compared to conventional FRET but is based on a similar mechanism [28]. For simplicity, the term FRET will be used to include LRET and FRET in this paper. The Ln ions absorb light poorly and require a sensitizer molecule for luminescence. The sensitizer molecule, (*N*-Methyanthranyl) will act as the antenna absorbing light and transferring that energy to the Ln ion.

### 2.2. Synthesis and Photophysical Properties

**Figure 2.**
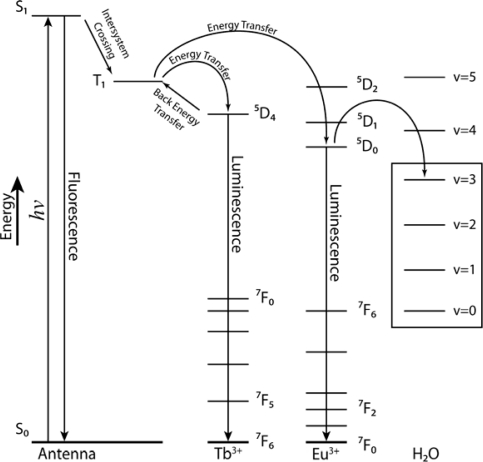
Electronic energy level diagrams for Tb^3+^ and Eu^3+^. Radiationless energy transfer competes with the radioactive process through coupling of the emissive states to the O-H vibrational overtones of the coordinated water molecules.

Three Ln(III) receptors have been prepared (compounds **3a**, **3b** and **4**, Scheme 1) adapted from known literature procedures [29,30]. In order to test whether a MA-DFB (**2**) ligand can act as an antenna to sensitize the Eu(III) metal center, compound **3a** was synthesized and isolated by reacting MA-DFB (**2**) with EuCl_3_•6H_2_O with triethylamine and the Eu(III). Europium(III) complexes typically show an intense luminescence signal between 610 and 620 nm [31]. The energy transfer (triplet state) from the ^3^An-Ln to An-Ln* (An = antenna) can occur *via* two mechanisms; the Dexter mechanism or the Förster energy transfer mechanism. The formation of the lanthanide excited state (Ar-Ln*) is a reversible process, and luminescence is dependent on a number of criterion; (1) how well the T_1_ excited state is populated, (2) The energy difference between the excited state of the antenna and the ^5^D excited state of Ln *i.e.*, is the energy gap large enough such that the lanthanide emission cannot be quenched *via* back transfer to the antenna triplet state (^3^Ar-Ln), (3) The distance between the antenna and the lanthanide which follows a *r*^6^ dependence, and (4) The number of coordinated water molecules, as the fourth overtone of the H_2_O oscillator is lower in energy than the ^5^D state, and therefore decrease the quantum yield of the metal luminescence, (figure 2; boxed in region). Unfortunately, no luminescence emission was observed for compound **3a**. The choice of antenna is very important in the receptor design, the lowest excited states of Eu(III) and Tb(III) are 17,200 cm^−1^ and 20,500 cm^−1^, respectively, due to point (2) mentioned above. For efficient population to the lanthanide excited state the energy of the triplet excited state needs to be at least 1,700 cm^−1^ above the excited state of the lanthanide ion, to prevent a thermally initiated back energy-transfer process, from An-Ln* to ^3^Ar-Ln and hence a signal response. If, however, the energy gap is less than 1,500 cm^−1^, such thermally activated back-energy transfer competes to repopulate the triplet state of the antenna and as a consequence no lanthanide luminescence is observed.

**Figure 3.**
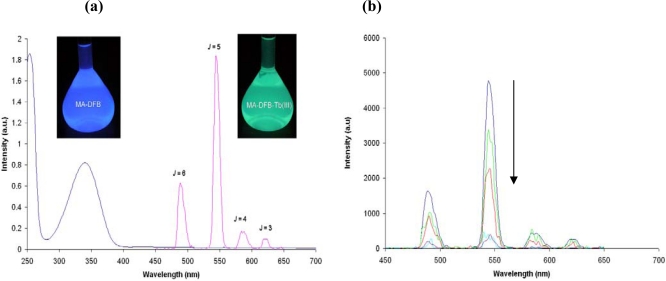
(a) The UV-Vis (340 nm) and the corresponding Luminescence spectrum of compound **3b** (Ex = 340 nm) in ethyl acetate (1 × 10^−8^ M) and (b) The UV-Vis (340 nm) and the corresponding decreasing Luminescence of compound **3b** (Ex = 340 nm) in ethyl acetate (1 × 10^−8^ M) with increasing amounts of FeCl_3_ added.

Therefore we switched the lanthanide ion to Tb^3+^ and prepared compound **3b.** Upon the excitation of compound **3b** at 340 nm, four distinct luminescence emissions are measured (transitions are ^5^D_4_-^7^F_J_ whereby *J* = 6,5,4 and 3) (also seen visually) in organic solvents such as ethyl acetate (Figure 3a). The initial studies showed that luminescence emission for compound **3b** was seen in a variety of solvents (ethyl acetate (Figure 3a), acetone, dichloromethane, chloroform, acetonitrile, and diethyl ether) and we have demonstrated that the fluorescence is quenched upon the addition of FeCl_3_, (ethyl acetate Figure 3b). However, like the Eu(III) complex, the luminescence of the Terbium is quenched by protic polar solvents such as MeOH and solvents that are hydroscopic, for example DMSO, when water occupies the vacant metal coordination sites (compounds 3a and 3b).

**Figure 4.**
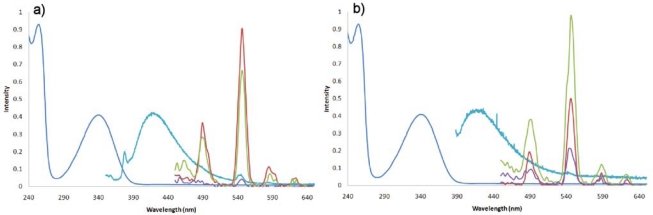
**(**a) The spectra showing absorbance (dark blue 100% MeOH, compound **2**), steady-state (light blue100 % MeOH, compound **3b**) and gated luminescence (red 100% MeOH (**3b**), green 50:50% MeOH:H_2_O (**3b**), purple 100% H_2_O (**3b**)). (b) The spectra showing absorbance (dark blue 100% MeOH, compound **2**), steady-state (light blue100 % MeOH, compound **4**) and gated luminescence (red 100% MeOH (**4**), green 50:50% MeOH:H_2_O (**4**), purple 100% H_2_O (**4**)). Conditions: Ex 340 nm, Em 350–650 nm, slit width 2.25 mm, delay 200 μs.

In order for our system to work as a sensor in oceanographic applications, it is essential that the signal response is not quenched by water molecules. This would require a luminescence signal in solvents such as methanol (MeOH), ethanol (EtOH) or dimethyl sulfoxide (DMSO), organic solvents that are miscible with water. In this paper we demonstrate that indeed a luminescence signal is observed for compound **3b**, in 100% MeOH and a 50:50% MeOH:dH_2_O mix (Figure 4a). As noted, the luminescence signal is quenched in 100% dH_2_O; because O-H, (like C-H moieties), is a higher-energy oscillator (*vide supra*) it significantly quenches the Ln luminescence, (Figure 4a purple line). We therefore modified the design of our molecular receptor to incorporate a “blocking” ligand that would prevent water molecules from coordinating to the Tb(III) centre. β-Diketonates have been used as sensitizers for lanthanide complexes [29,32–35]. β-Diketonate ligands coordinate via the two oxygen atoms leading to neutral species in a 3:1 β-diketonate:lanthanide ratio, the complexes have been shown to be stable in aqueous solutions [35]. Therefore these molecules have been used in many applications from sensing [36], antibody labeling [37], new materials such as liquid crystals [38], near IR-LEDs, [39] sol-gel glasses [40], and polymers [40].

Incorporating a commercially available β-diketonate, for example, 1,1,1,5,5,5-hexafluoro-2, 4-pentanediione (Hhfac) ligand into our molecular receptor we synthesized compound **4**, (Scheme 1). Again the same luminescence experiments were carried out in 100% MeOH, 50:50% MeOH:dH_2_O and 100% dH_2_O (pH 8) and in all three solvent systems a luminescence signal, is observed, (Figure 4b).

**Figure 5.**
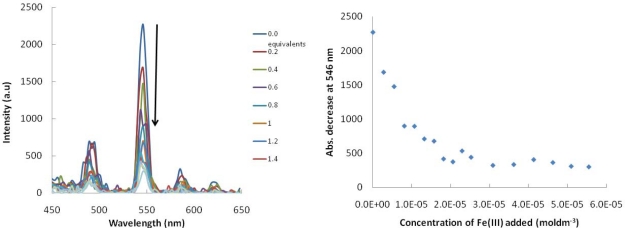
Fluorescence titration with **4** and FeCl_3_ (0 to 5 equivalents) MeOH:H_2_O (50:50). RHS: Binding isotherm obtained showing the decrease in absorbance at 546 nm on the addition of Fe(III).

We have shown that the displacement of the lanthanide with FeCl_3_ is achieved in organic solvents, ethyl acetate, (Figure 3b). The same titration experiment has been carried out with compound **4** and FeCl_3_ in MeOH and dH_2_O (50:50%) and the same trend is seen, the luminescence is quenched rapidly upon the addition of small aliquots of Fe^3+^. The concentration of the Fe(III) equals 2.5 × 10^−5^ moldm^−3^ at the point where there is no more quenching (Figure 5).

This preliminary data provides us with a good understanding of the system and evidence that this theoretical approach will work for development into an iron chemosensor. This data clearly demonstrate that with a slight modification of the molecular receptor, one can observe luminescence in 100% dH_2_O and that this luminescence decreases as a function of increasing [Fe(III)]. Our initial studies provide an effective detection limit, based on the precision of the luminescent signal as low as 5 nM (assuming a 1L sample), an environmentally relevant concentration.

## 3. Experimental Section

**Materials.** Chemicals: All chemicals were purchased from Sigma-Aldrich Chemical Co. (St.Louis, MO) and used without further purification. N-methylisatoic anhydride was purchased from TCI America. Solvents used were all of UV-spectroscopic grade and purchased from Sigma-Aldrich, or VWR.

^1^H and ^13^C NMR spectra were recorded on a Bruker UltraShield plus 400 MHz spectrometer in DMSO-*d*_6_. Chemical shifts are reported in parts per million (*d*) downfield from tetramethylsilane (0 ppm) as the internal standard and coupling constants (*J*) are recorded in Hertz (Hz). The multiplicities in the ^1^H NMR are reported as (br) broad, (s) singlet, (d) doublet, (dd) doublet of doublets, (ddd) doublet of doublet of doublets, (t) triplet, (sp) septet, (m) multiplet. All spectra are recorded at ambient temperatures. IR was taken using a Nicolet Nexus 470 FT-IR paired with a Smart Orbit ATR attachment, with the characteristic functional groups reported in wavenumbers (cm^−1^). UV-vis experiments were performed on a Beckman DU-70 UV-vis spectrometer. A stock solution (2.9 × 10^−4^ moldm^−3^) of compound **2** was prepared by dissolving 2.0 mg in 10 mL of MeOH and 1 mL transferred to the UV-vis cell. To which 2,4,6 trimethylpyridine (1μL, 26 equivalents) was added to compound **2**. A second stock solution of TbCl_3•_6H_2_O salt was prepared by dissolving 7.1 mg of Tb^3+^ salt in 5 mL of MeOH (3.8 mmol), from the Tb^3+^ salt stock solution a 2.9 × 10^−3^ mol dm^−3^ solution was prepared by transferring 152 μL to a 2 mL cell. Aliquots of the Tb^3+^ was then added incrementally up to 10 equivalents. Fluorescence experiments were carried out on a QuantaMaster^™^ 40 Intensity Based spectrofluorometer; steady-state (slitwidths 1.00 mm); λ_Ex_ = 340 λ_Em_ = 360 to 600 nm and gated emission (slit widths 3.0 mm, delay time 200 μs, λ_Ex_ = 340 λ_Em_ = 450 to 650 nm). MA-DFB and the Lanthanide salts were diluted to 2.25 × 10^−6^ moldm^−3^ and 2.2 × 10^−4^ moldm^−3^ respectively and used for the fluorescence studies. A FeCl_3_ solution (2.2 × 10^−4^ M) was prepared in MeOH and aliquots of 10 μL was titrated into the UV-vis or fluorescence cell. The dilution was taken into consideration when calculating the equivalents of host:guest solution.

**Synthesis.** Compound **2** (MA-DFB): The synthesis of MA-DFB was adopted from Loyevsky *et al*. [41]. Desferrioxamine (330 mg, 0.5 mmol) was dissolved in dimethylformamide (1 mL) and triethylamine (73 mg, 0.730 mmol). The solution was allowed to stir for 15 minutes, to which *N*-methylisatoic anhydride (90 mg, 0.5 mmol) was added to it, the resulting mixture allowed then to stir overnight at STP under argon. The supernatant was removed by centrifugation and the white solid was washed several times with ddH_2_O, then washed with diethyl ether and dried in vacuum for 24 h. Melting point: 177–181 °C; UV (methanol): 340 nm (ε = 2398 M^−1^ cm^−1^) and 250 nm (ε = 4659 M^−1^ cm^−1^); IR (neat ATR): 3304 (NH amide), 3092 (OH oxime), 1617 (CO-N) cm^−1^; ^1^H-NMR (DMSO-*d*_6_): 9.65 (br, s 3H OH oxime); disappears on D_2_O shake at room temperature, 8.27 (t, 1H, NH amide); disappears on D_2_O shake at 60 °C, 7.80 (m, 2H, NH amide) disappears on D_2_O shake at 60 °C, 7.61 (m, 1H, 2° NH amine); disappears on D_2_O shake at room temperature, 7.50 (m, 1H, ArH), 7.30 (m, 1H, ArH), 6.60 (m, 2H, ArH), 3.49 (m, 6H, CH_2_), 3.17 (m, 2H, CH_2_), 3.00 (m, 6H CH_2_), 2.75 (m, 4H, CH_2_), 2.55 (m, 4H, CH_2_CH_2_), 2.25 (m, 4H, CH_2_CH_2_), 2.34 (s, 3H, NCH_3_), 1.95 (s, 3H COCH_3_), 1.50–1.23 (12H, CH_2_CH_2_CH_2_); ^13^C-NMR (DMSO-*d*_6_): 172.5, 172.0, 170.7, 169.8, 150.2, 132.8, 128.7, 115.9, 114.4, 110.0, 49.1, 47.6, 47.3, 38.9, 30.3, 29.8, 29.3, 28.0, 27.2, 26.5, 26.2, 24.0, 23.3, 20.9 ppm.

## 4. Conclusions

We describe a novel approach that utilizes an indicator displacement assay to develop a chemosensor for the biologically available iron fraction in aquatic systems. This approach combines TRF and FRET with the unique photophysical properties of a lanthanide ion to increase the sensitivity of a fluorescent siderophore derivative, MA-DFB. The siderophore is derived from a natural siderophore, desferrrioxamine B, a strong fungal chelator. Currently there are very limited means to measure iron that is biologically available to eukaryotic phytoplankton. The development of this chemosensor will help build a better understanding of this fraction of iron in marine systems. The capability of measuring labile iron *in situ* with a photoactive chemosensor will lead to a more comprehensive understanding of primary production and the ocean carbon cycle.
